# The enduring flame: our legacy of resilience, a time of unprecedented potential, and igniting the future of discovery

**DOI:** 10.1172/JCI199593

**Published:** 2026-01-02

**Authors:** Anna Greka

**Affiliations:** Harvard Medical School, Mass General Brigham, Boston, Massachusetts, USA, and Broad Institute of MIT and Harvard, Cambridge, Massachusetts, USA.

Esteemed colleagues, honored guests, and fellow members of the American Society for Clinical Investigation, it is a privilege to stand before you today, having had the honor of serving this Society for the past 6 years as Councilor and now as President.

As we gather today, I find myself reflecting on our Society’s rich 117-year history, looking to gain insights into how we can confront the unique challenges and opportunities of our present moment, and chart a course for the future. My talk is therefore organized in 3 chapters, representing the past, present, and future of our organization.

The essence of my message today centers on the enduring flame of discovery, founded on a legacy of resilience and innovation, that continues to ignite our present and light a path to our future.

## I. The past: a legacy forged in resilience

The ASCI was founded in 1908 on the principles of scientific rigor, collaboration, and a relentless pursuit of knowledge for the benefit of patients, who are the ultimate stakeholders for everything we do as physician-scientists. Over these last 117 years, the organization has been tested time and again. We have navigated periods of profound upheaval — world wars that reshaped the global landscape, economic depressions that threatened the very foundations of our society, and numerous crises that demanded our immediate and unwavering response.

As the first ASCI president of Greek heritage, I am particularly mindful of this legacy of resilience. My own ancestors carried the flame of hellenic thought and inquiry through centuries of challenge, including the age of Alexander the Great. Alexander is especially significant to me because I was born in Thessaloniki, a city he named in honor of his sister. Speaking of Alexander, he was in fact a student of another “Great,” the philosopher and scholar Aristotle the Great, who lived and worked just a few miles from where I was born. In my mind, it is Aristotle’s works that best exemplify the hellenic spirit of scientific inquiry, of discovery, and the concept of *philosophia,* the love of knowledge.

While we are on the topic of Greek words, there is another principle that I believe is equally important to the love of knowledge for a physician-scientist: *philotimia*, or love of honor. I would like to think that love of knowledge and love of honor are deeply woven into my own identity and my commitment to my career as a physician-scientist — and of course, these values are also woven into my commitment to this organization. As I stand here today with profound gratitude, I am cognizant that my presence is in itself a testament to the fact that the pursuit of knowledge in our honorable profession knows no borders and unites us all.

As a student of the ASCI’s history, I recently learned of the challenges faced by our predecessors. In the shadow of the First World War, which the United States formally entered in 1917, the number of students enrolled in US medical schools drastically declined. George Blumer of Yale University devoted his 1918 ASCI presidential address to sharing his profound concern over threats to the “very existence of the [medical] schools,” as he put it, and the risk that the war would diminish their newly found “investigative spirit” (1).

Decades later, in 1952, in his presidential address, Barry Wood Jr. of Washington University in St. Louis contemplated the possible effects on medical science of atomic warfare, of a sudden collapse of the world economy, of a sudden drying up of sources of research funds. In his own words: “Any one of these and a host of other unpleasant eventualities might cause a significant deceleration of medical progress even within our own lifetimes” (2).

Does any of this sound familiar? I do not share these stories as mere anecdotes of resilience in the face of profound challenges. Learning about our Society’s rich history reminded me, and I hope it will remind all of you, that even in the darkest of times, the pursuit of knowledge and the commitment to our honorable mission must prevail — and have indeed prevailed over 117 years.

Through each trial, the ASCI has emerged stronger, its core values reaffirmed, its commitment to rigorous science in service of patients deepened. This resilience is the enduring flame that connects us to our past and guides us forward.

## II. The present: a time of unprecedented potential and profound responsibility

Turning to the present, I believe we are in the midst of a biomedical revolution. The convergence of genomics, advanced imaging and spatial technologies, CRISPR and gene editing tools coupled to the unprecedented power of machine learning and artificial intelligence — what I collectively call “biology at scale” — has ushered in a golden era of discovery.

I believe that this remarkable revolution allows us to reaffirm one of the founding principles of our Society: the study of patients, of human beings. With all our modern tools and technologies, we are now better armed than ever to gain systematic insights into human physiology at a deep molecular level.

**Figure 1:**
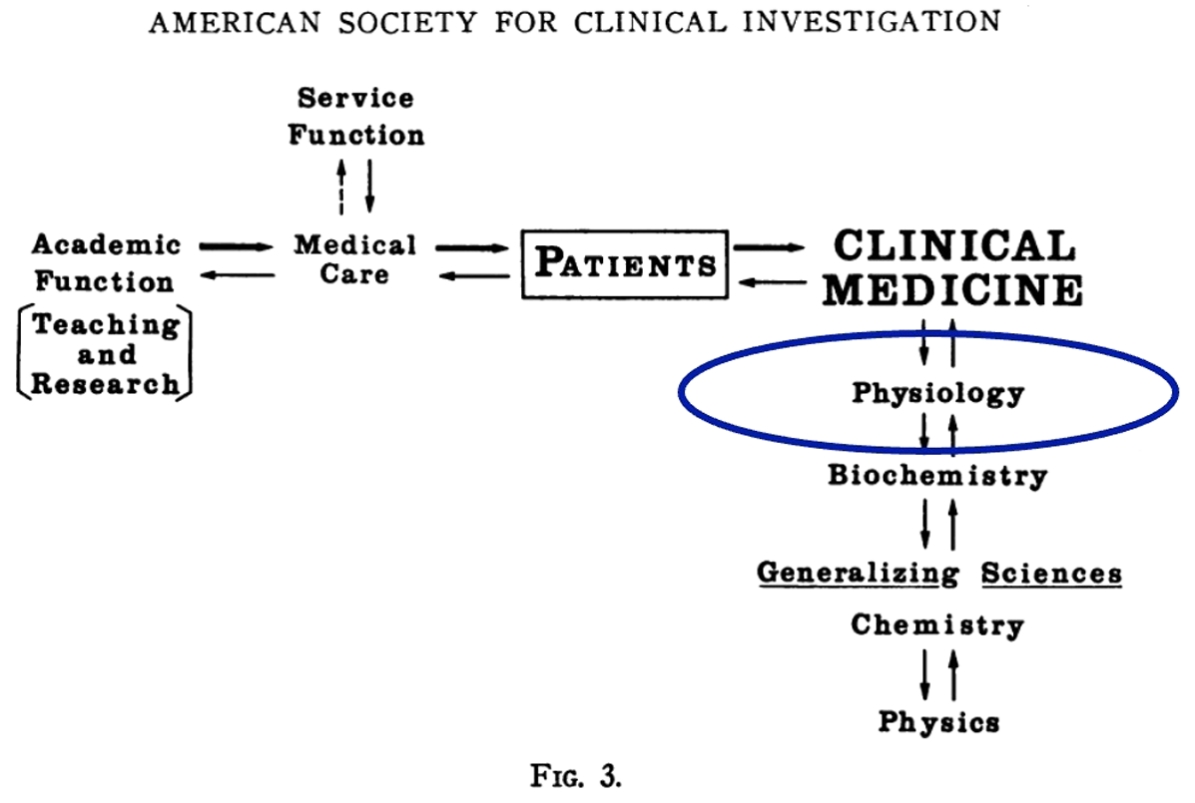
Figure from Donald Seldin’s 1966 ASCI presidential address. Adapted from ref. 3.

I cannot help but think that Donald Seldin — a legendary president of this Society, the father of modern nephrology, and a personal hero of mine — would be pleased to know that Figure 3 from his presidential address in 1966 (Figure 1; ref. 3) remains highly relevant today! In other words, there has never been a better time to be a physician-scientist.

I often liken our work to climbing because our path to treatments and cures is hard, nonlinear, and frequently requires stopping, regrouping, and getting back to the climb with renewed vigor. And I believe our efforts are being rewarded: we are living in a golden era, having at our disposal therapeutic modalities that just a few short years ago would be considered unimaginable.

As we will hear from George Yancopoulos tomorrow, human antibodies produced at scale have revolutionized the treatment of devastating diseases. Also in our program tomorrow, we will celebrate our immense progress in treating genetic diseases like cystic fibrosis, as told by Michael Welsh and our first ASCI/Scharschmidt~Crawford Award recipient, David Altshuler.

**Figure 2:**
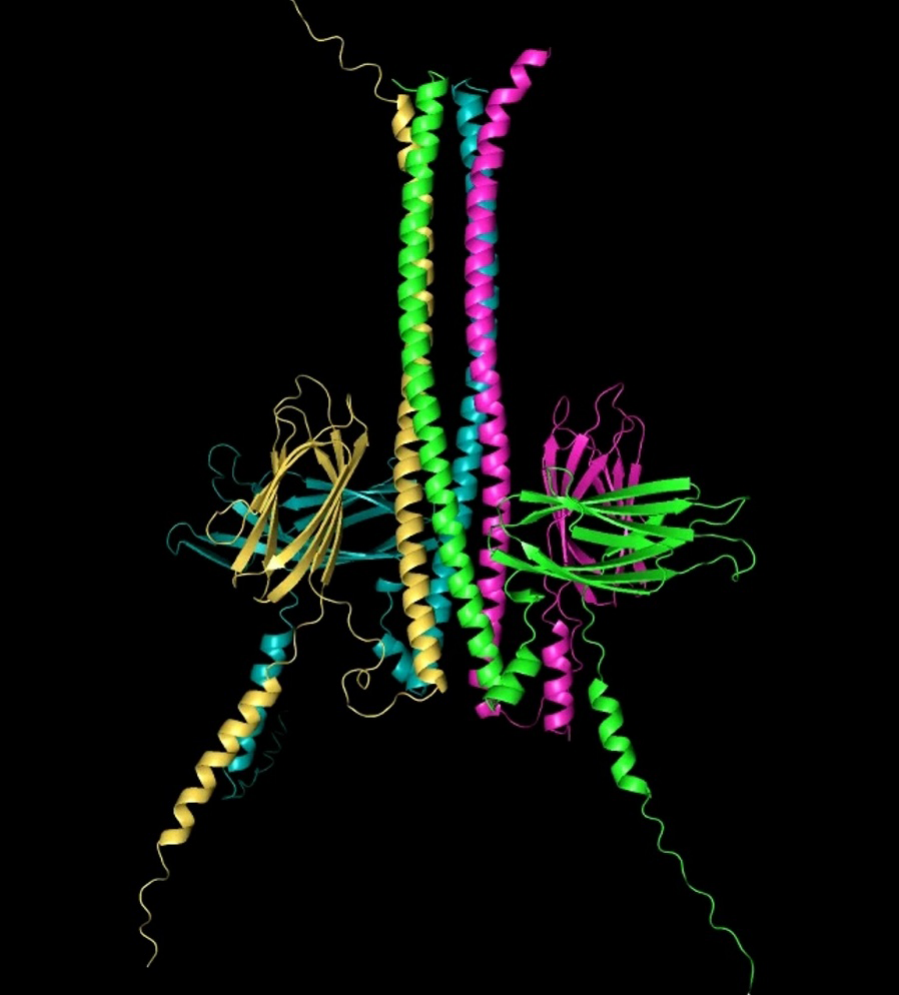
AlphaFold3 structure of a heteromeric protein complex.

And if you will indulge me for a moment, here is an AlphaFold structure of a heteromeric protein complex that my lab is working on. It is the target of a newly developed drug for which we have high hopes, as it appears to have preclinical efficacy across several genetically defined proteinopathies in the kidney, the eye, and the brain (Figure 2). My point here is that AlphaFold has massively accelerated our efforts, considering that the confirmatory cryo–electron microscopic structure for this target has taken us years to obtain.

All these breakthroughs — antibody therapies, genetic disease treatments, and more — were driven by decades of fundamental research, followed by innovative approaches to clinical translation. And this entire climb from discovery to final approval was in every instance guided by physician-scientists. Again, there has truly never been a better time to be a physician-scientist.

Yet this era of unprecedented potential is shadowed by a growing threat: the corrosive influence of political polarization and the erosion of trust in science. But here’s a fundamental truth: Our work is not political. Health is a matter that affects each and every one of us. A recent poll commissioned by the nonpartisan organization Research!America confirms that health and medical progress matters to all Americans, regardless of beliefs or affiliations (4). Our common humanity, our common mortality make our work as physician-scientists relevant and essential for all.

As such, our mission, our North Star if you will, is our patients. Let us partner with our patients as their advocates, their champions, and their hope. Let’s strengthen our ties with patient advocacy groups, ensuring that their voices are heard. Let’s communicate the value of our work to the public, bridging the gap between the laboratory and the bedside with honesty, clarity, and conviction. Policies that support robust and sustained funding for biomedical research benefit our economy, ensure our leadership on the world stage, and secure a future of good health and prosperity for all Americans.

## III. The future: igniting the next generation of discovery

Speaking of the future... Our future rests in the hands of our young colleagues — the brilliant minds who are pushing the boundaries of knowledge and embracing new frontiers. When I joined the ASCI Council 6 years ago, I made supporting our younger colleagues the focus of my efforts.

Few organizations are like ours, singularly focused on the critical role of physician-scientists, making the case that our profession is essential to the US biomedical enterprise. Beyond our leadership at academic centers, over the last 4 decades, physician-scientists constituted more than 70% of chief scientific officers at top biopharmaceutical companies. We are lucky to have many of our colleagues from these companies here, at this meeting, and I would like to extend a warm welcome to them, to all of you, as we all serve the same mission and purpose.

**Figure 3:**
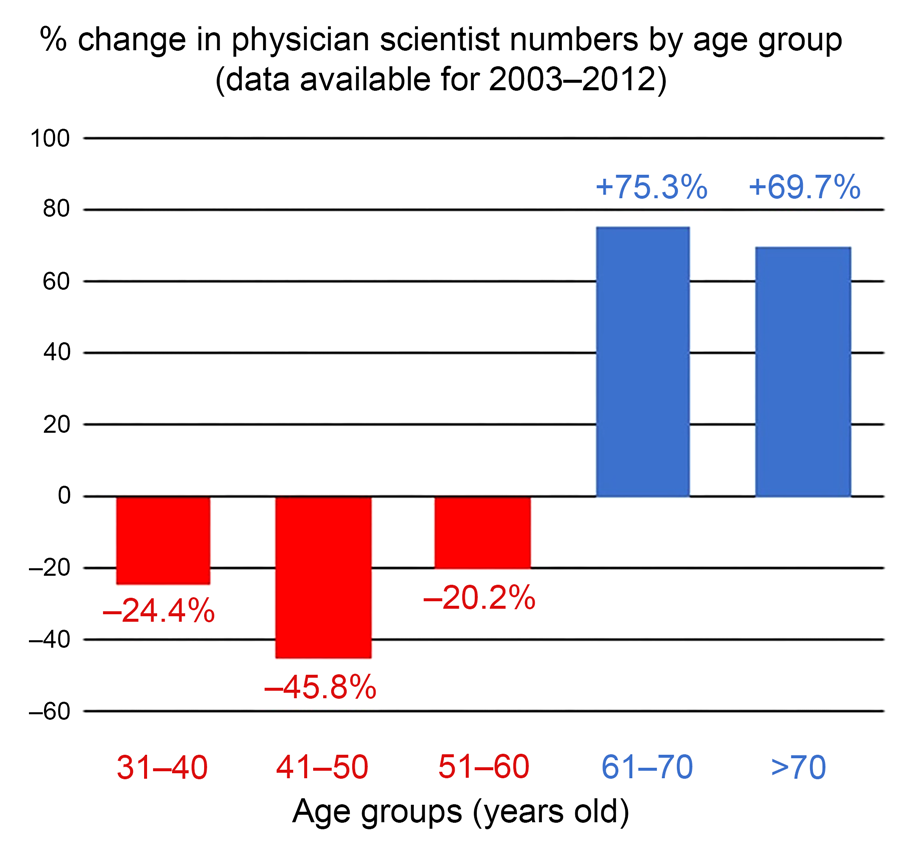
Change in US physician-scientist numbers, 2003–2012. Adapted from ref. 5.

Concerningly, and this will not come as a surprise to any of you, the physician-scientist workforce in the United States has both declined and aged over the last decades. As this chart from NIH data that is admittedly now a decade old shows (Figure 3) (5), significant attrition at early career stages renders our pipeline vulnerable to collapse. Since these data were collected, it is estimated that the physician-scientist workforce has diminished even further, now constituting less than 1% of all physicians in the United States (6).

**Figure 4:**
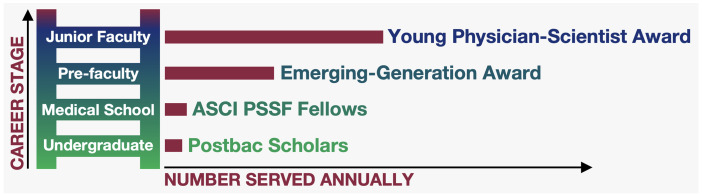
ASCI early-career programs, 2024–2025.

To specifically address the attrition at the early stages of our pipeline, the ASCI has piloted several programs (Figure 4). Some of these programs are directed to students, aiming to provide research training via the ASCI Postbaccalaureate Program and the ASCI Physician-Scientist Support Foundation (PSSF) Fellowships for medical students. For colleagues at the very vulnerable late-postdoctoral and pre-faculty stage, we established the Emerging-Generation (E-Gen) Awards, complementing our longer-running early-faculty Young Physician-Scientist Award (YPSA) program. Perhaps most important of all is the proverbial “tap on the shoulder” that our younger colleagues receive when selected for these competitive programs. In our annual surveys, we hear that they gain confidence from this recognition — and in a few short years, our success is measured by the high rate of conversion from some of these early-career programs to full ASCI membership. In other words, these programs are shoring up our pipeline.

Now, as you all know, the global landscape of biomedical research is becoming increasingly competitive. To ensure that the United States remains a leader in this critical endeavor, we must invest strategically in the success of our younger colleagues. To this end, it is my honor to announce the ASCI Impact Fund, whose goal is to endow our early-career programs in perpetuity. I invite all of you to contribute generously to the Impact Fund. Let us empower our young colleagues to be bold, to be innovative, and to be fearless in the pursuit of their career paths. Let us equip them with the tools they need to make the breakthroughs that will define the next century of biomedicine.

In closing, I would like to return to the beginning and acknowledge once again what an immense honor it has been to serve this Society over the past 6 years. I am indebted to all my colleagues on the Council who have been tremendous partners on this journey. The ASCI staff are the heart and soul of the Society, and they approach their work with great professionalism and a sense of mission. In addition to John Hawley and Karen Guth, who have safeguarded our organization for decades, I would like to especially acknowledge Colleen McGarry, whose passion and dedication to our early-career programs are the key to their ongoing success. Finally, the Society is in excellent hands, as I am honored to pass on the torch to Priscilla Hsue. who will guide the ASCI with *philosophia* and *philotimia*.

Speaking of these profound hellenic values that go back thousands of years, I am grateful to my parents, who taught me these values early on, not only with their words, but also with their actions. And last but certainly not least, I must acknowledge my better half, Peter Mundel, who is also an ASCI member. What I am most grateful for is our partnership — Peter is my true best friend on our journey navigating our busy careers and lives as physician-scientists.

In conclusion, the ASCI stands at a critical juncture. We are heirs to a legacy of resilience and innovation. We are called to action in a time of unprecedented potential and profound responsibility. And we are entrusted with igniting the future of discovery. Let us rise to this challenge with courage, conviction, and an unwavering commitment to the patients we serve. Let the enduring flame of the ASCI guide us forward. It’s up to us — all of us.

## Version 1 01/02/2026

Electronic publication
